# Kinect-Based Virtual Game for the Elderly that Detects Incorrect Body Postures in Real Time

**DOI:** 10.3390/s16050704

**Published:** 2016-05-16

**Authors:** Zelai Saenz-de-Urturi, Begonya Garcia-Zapirain Soto

**Affiliations:** DeustoTech Institute of Technology, DeustoTech-LIFE Unit, University of Deusto, Bilbao 48007, Spain; mbgarciazapi@deusto.es

**Keywords:** Kinect, postural control, virtual game, elderly, dynamic time warping algorithm

## Abstract

Poor posture can result in loss of physical function, which is necessary to preserving independence in later life. Its decline is often the determining factor for loss of independence in the elderly. To avoid this, a system to correct poor posture in the elderly, designed for Kinect-based indoor applications, is proposed in this paper. Due to the importance of maintaining a healthy life style in senior citizens, the system has been integrated into a game which focuses on their physical stimulation. The game encourages users to perform physical activities while the posture correction system helps them to adopt proper posture. The system captures limb node data received from the Kinect sensor in order to detect posture variations in real time. The DTW algorithm compares the original posture with the current one to detect any deviation from the original correct position. The system was tested and achieved a successful detection percentage of 95.20%. Experimental tests performed in a nursing home with different users show the effectiveness of the proposed solution.

## 1. Introduction

Poor posture may cause physical problems such as back pain, spinal dysfunction, joint degeneration, and muscle fatigue *etc.*, including the risk of loss of balance and falls, especially in senior citizens. Bad posture over many years can result in poor physical function [1].

Good posture becomes even more important when the subject is prevention, maintenance, or recovery of health or corporal well-being. Scientific articles [2,3] disclose that 80% of the population is affected at some time in their lives by backaches that emphasize the need for good posture in order to guarantee or recover the body’s proper functioning. Hospitals and schools suggest programs to educate body posture which, based on biomedical knowledge, aim to teach correct postures to treat or prevent pain-related problems [4,5]. Maintaining a good physical functional ability is an important prerequisite for preserving independence in later life. In fact, research has shown that being physically active is associated with less morbidity and mortality [6,7,8].

In general, a physically active lifestyle should be promoted to maintain good health, bearing in mind that certain populations are at particular risk of poor physical function and could potentially benefit from early intervention. However, long-term maintenance of physical activity is a challenge, because lifestyle habits and barriers to physical activity are often ingrained in the older population [9].

A recent study conducted by Allaire *et al.* [10] suggests that playing video games may serve as a positive activity associated with successful ageing. In addition, virtual reality applications could encourage to increased physical activity in elderly persons. A recent review of virtual reality applications used to improve health shows positive progress towards adapting new technology within a specialized health context. Most health game studies include physical activity (28%) with what are referred to as exergames (serious games to improve physical exercise) [11]. A meta-analysis of energy expenditure in exergaming shows that playing exergames significantly increased heart rate and oxygen uptake [12]. Serious games create stimulating methods to maintain an active lifestyle tailored to the specific physiological and psychological requirements of the elderly and disabled while providing the benefits of physical exercise routines [13]. This type of video game includes interfaces that allow games to be played from almost any position: sitting, standing, or even prone. This is possible because the hand-operated devices are based on buttons and thumb-operated levers. A game designed to promote physical activity must be equipped with technology that detects the general motion of the human body. Thus, therapists have started experimenting with exergames in their work, using low-cost devices that detect human motion, such as the Nintendo Wii^®^ or Microsoft Kinect. Many applications related to elderly care that use these devices can be found in literature [13,14,15]. However, of these two devices, Kinect is the most feasible technology for a widely-used system of elderly exergaming as it utilizes vision-based data capture and requires no extraneous hardware, such as a game controller [13].

Kinect is a motion sensor, and even with some inconsistences in the motion capture [16], Clark *et al.* [17], Obdrzálek *et al.* [18], and Fernandez-Baena *et al.* [19] concluded that, in general, the Kinect has sufficient accuracy for the assessment of whole-body kinematics for postural control and diagnostic purposes.

Therefore, the aim of this research is to detect incorrect postures while the elderly are performing physical activities through a virtual 3D exergame in real time. A long term objective of the system is to encourage users to learn about their postural control. To achieve this, the system uses the Kinect sensor to capture users’ movements and give them the feedback needed about their corporal postures. The system’s algorithm compares the user’s original posture at the beginning of the game with the current one in order to detect any deviation from the original correct position.

The rest of the paper is organized in this manner: the following section presents the background in this field. The proposed Corporal Posture Correction System and the Game System Overview are explained in Section 3. Section 4 discusses the results obtained from the study and Section 5 draws the conclusions about the system presented and the future work.

## 2. Background

Human body part detection and tracking have been widely studied in literature on the subject. In the past, camera-based motion capture systems that required cumbersome markers or suits were used whereas recent research has focused on markerless camera-based systems. The complexity of such systems regarding image processing depends largely on how the scene is captured. When 2D cameras are used, problems such as the variety of human motions, occlusions between limbs or with other body parts, and sensitivity to changes in light are difficult to cope with. These problems can be solved with a capture system that provides depth information, such as multi-camera systems or binocular cameras. Another type of depth camera that appeared on the market recently is the time of flight (TOF) camera, which uses an infrared light beam to illuminate the scene and then measures the phase lag between the waves sent by the transmitter to the receiver device. TOF cameras are very precise but require complex hardware, are expensive, and provide low resolution (160 × 120, e.g.,) [20].

As a low-cost alternative, the Kinect 1.0 sensor add-on for the Microsoft Xbox 360 game console was released at the end of 2010. It includes a structured light camera with a conventional RGB camera that can be calibrated to the same reference frame. The Kinect device interprets the 3D information of the scene obtained through infrared structured light that is read by a standard CMOS sensor.

In the summer of 2014, Microsoft launched a new generation of sensors on the market—Kinect 2.0, also known as Kinect for Xbox One—which has the same number of sensors as the Kinect 1.0. However, depth is measured with a completely different principle than the coded-light patterns used by the original one, providing the Kinect 2 with TOF technology. RGB images are acquired in high definition (1080p) and the sensor has a 60% wider field of view. The colored image of the user is obtained by this camera. In the research conducted by Pagliari *et al.* [21], the superior performance of the Kinect 2.0 was quite evident. Furthermore, the Kinect 2.0 provides a 25-joint skeleton model, comparing with the 20-joint model given by the Kinect 1.0.

The Kinect sensor is the leading markerless movement tracking tool, in a very affordable package. The skeletal tracking algorithm and the constantly updated software development kit (SDK) have led to the success of the Kinect. Users do not have to wear any sensors and interaction with the computer can be achieved through various gestures and postures.

Despite the fact that the Kinect sensor was originally designed to provide new types of games and improve the leisure experience for consoles such as the Xbox 360 game console, it has also been used in areas such as robotics, medicine, 3D reconstruction, and augmented reality, *etc*. Through virtual environments, many studies [22,23,24] have shown its potential for assisting recovery from illnesses such as strokes and vestibular disorders and improvements in cerebral palsy or balance. Virtual reality provides immediate feedback to the user, which enhances learning and also provides an automated way to guide and track the progress of the training [25].

A growing number of studies have explored ageing and the acceptability of physical exercise applications and virtual games using Kinect sensor or camera-based systems. More specifically, they found significant positive impact on mood while undertaking sequences of physical activities [26] and more physical cooperation between generations of users [27]. Positive findings have also appeared in the area of rehabilitation and physiotherapy: A medium-term study conducted by Smeddinck *et al.* [28] integrated into a normal rehabilitation program indicates an increase in motivational aspects such as autonomy or presence.

The study by Tang *et al.* [29] uses multi-camera views to guide people through pre-recorded physiotherapy exercises in the home. Their research also provides suggestions for exercise guidance systems drawn from qualitative findings on visual feedback complexity.

Suárez *et al.* [30] developed a vestibular rehabilitation system using virtual reality to recreate environmental changes. The findings show an adaptation in the postural control parameters after rehabilitation and suggest that virtual reality could be a tool for designing customized vestibular rehabilitation programs to prevent instability and falls in the elderly population.

Additionally, as a means to detect incorrect postures based on non-invasive technologies, Worthington presents his *PostureMinder* [31] as a commercial tool to help computer users to correct their posture. It compares a user’s defined seating position from which it alerts the user when it detects deviation. It uses the computer web-cam in order to record the users’ body using image processing techniques. Image processing has become a powerful tool for markerless body and head tracking, which is why the Kinect sensor is being widely used in applications ranging from medical and physical to 3D user interfaces.

Furthermore, a large number of applications explained in the literature use gesture recognition techniques to detect correct or incorrect postures based on hands or the whole body. In this area, the most widely used algorithm studied is Dynamic Time Warping (DTW) [32,33].

Other methods include Hidden Markov Models (HMM) [34,35], as well as Neural Networks (NN) [36]. Comparatively, DTW performs more accurately on a small set of gesture sequences, although it is limited by its computational demands when a larger set is used. Meanwhile, HMM and NN models require a more complex training phase, but the recognition pipeline has lighter computational demands. The rule-based approach to gesture recognition is also commonly used for the assessment of rehabilitation exercises such as in the research by Zhao *et al.* [37]. This approach does not require the recording of initial gestures and is less computationally intensive than the DTW-based method [38]. However, the DTW approach gives better accuracy for gesture recognition than the rule-based method, which is necessary for physical activity intervention systems. Furthermore, DTW-based methods are suitable for recognizing simple actions such as this case. If complex human actions are performed, the basic DTW is not considered to be the best approach.

In this work, we use the DTW algorithm to compare two sequences which have different time lengths in order to determine the similarity between the user’s original posture and the posture while playing.

## 3. Materials and Methods

### 3.1. Participants

Owing to the fact that the game is intended for elderly persons, the possibility should be taken into account that they may have certain disabilities as a result of their age that prevent them from playing it. Thus, the experiment was conducted with participants of different ages and with different disabilities—both physical and cognitive.

The authors, together with physiotherapists from the nursing home, set out the following criteria for including participants: They should have sufficient cognitive capacity to understand the game and the physiotherapists’ instructions, and in terms of physical condition, they should have minimum mobility in at least one arm. The cognitive level of each participant was measured using the MMSE (Mini Mental Status Examination) scale [39]: ≥23 = Normal; 22 – 20 = Mild; 19 – 11 = Moderate; ≤10 = Severe. Please refer to Table 1 for participants’ data.

In total, 15 users (10 women and 5 men) between 69 and 96 years of age (M = 83.6, SD = 7.56) took part in the experiment. Two of the participants had a visual deficit (presbyopia, an age-associated eye condition), two suffered from macular degeneration, three of them used wheelchairs for their daily activities and one suffered from Parkinson’s disease.

All of them—both those who used wheelchairs and those who were able to play standing up—were able to complete the experiment.

### 3.2. Experimental Design

Permission to conduct the tests was obtained from the Ethics Committee at the University of Deusto, as well as from the participants’ nursing home. This consent, written in Spanish (the participants’ mother tongue), was signed by all of them prior to commencing the study.

All participants were explicitly informed about each of the tasks that needed to be performed during the experiment, its purpose and the consequences deriving from the data obtained.

In order to prevent participants from any environmental distractions, the experiment was conducted in their habitual nursing home, thus ensuring that performance was not limited and enabling the same conditions for which the system had been developed. The physiotherapists who work with them on a daily basis were also present throughout the experiment to provide assistance if necessary.

Two game sessions were established with a six-month gap between them. Each participant played an individual game session of 9 min duration each (3 min per game level). All participants received instructions on how to play each level as well as how to set up the system. Once the game session had concluded, each participant was provided with a satisfaction questionnaire, and both sessions were recorded and observations made. Regarding the participants with mild cognitive impairment, required assistance was provided by the psychologists due to the complexity of the questionnaires.

The experiment was conducted by experts in different areas of physiotherapy, psychology, and IT.

### 3.3. Corporal Posture Correction System

In order to develop the Corporal Posture Correction system, the Microsoft Kinect SDK, an open-source library for testing and implementation, has been used. This library provides a 3D virtual skeleton which consists of the positions of 25 joints and body parts (such as the wrists, knees, head, and torso), from which a 3D virtual avatar is generated. The joints that are used during movement are mapped directly onto the values placed on the avatar puppet within the game world. Matching these two models is natural, so the system can capture a Kinect posture as well as monitor in real time whether or not a user posture matches the previously defined correct position.

**Figure 1:**
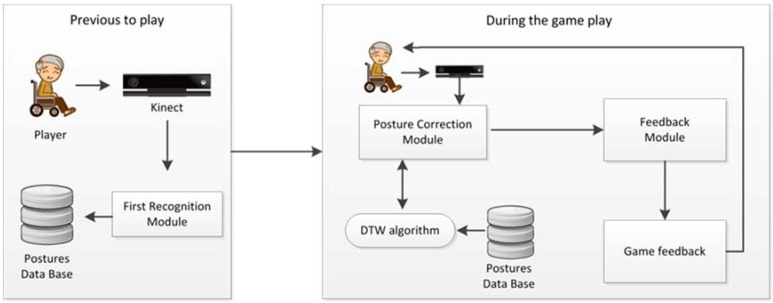
General diagram of the modules.

The Corporal Posture Correction System is aimed at correcting users’ bad posture while they are seated on a chair or standing up in front of the camera. It comprises various sub-modules: First recognition module, posture correction module, and feedback module. A general diagram is explained in Figure 1.

#### 3.3.1. First Recognition Module

The First Recognition Module involves taking the first image of the user and storing it in the game data base. The main idea is to obtain a complete gesture of the user, that is, a gesture starting with the back in a good initial position and the end, after tilting their back to a bad position. To achieve this, users must be sitting up straight in the chair or standing up, with their back, neck, and head aligned. In this case, not all the elderly could manage this position so they remain in a comfortable posture but one which is very similar to this. The user must stand about 1.5–2 m in front of the Kinect sensor, and maintain this position without moving for a few seconds. In order to take a better view of the user, the sensor must be adjusted by tilting it up and down. Unlike Kinect 1.0, which can be adjusted automatically, the Kinect 2.0 sensor can only be adjusted manually.

If the user is going to play while seated, the SDK provides a seated tracking option. However, in order to ensure greater accuracy in obtaining the user’s real position, this module also stores the three hip nodes. During the seated mode, the user’s back must always be in contact with the chair back for full support.

**Figure 2:**
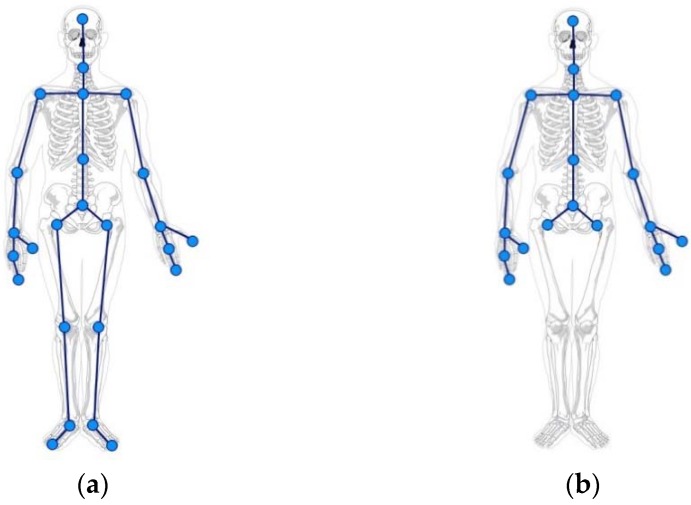
(**a**) 25 body components of the skeleton model; (**b**) 19 upper body components of the skeleton model for seated users.

The joints the Kinect 2.0 SDK’s provides are represented in Figure 2. Each joint or node has three coordinates: X, Y, Z.

When recording the gesture, the user has 10 s to get ready. After acquiring a comfortable position the users must tilt their back to a bad position as soon as the recording starts and finish it on the 32nd frame. This might mean that the user needs to perform the gesture for the recording slower (or more quickly) than he would do in real life. The DTW algorithm does not take into account how quickly the gesture is performed.

The system will display an alert to the user indicating that he has reached a bad posture (more than 20 degrees) and will stop the recording.

A position is considered bad when an angle of 20 degrees or more is reached. This position was established by the physiotherapists who work daily with the participants, taking into account their physical condition.

The First Recognition Module calculates the bad posture by checking if it has more than 20 degrees of deviation in relation to the back’s initial vertical position. After the recording, the system stores the gesture in an *xml* file with all the coordinates labelled for further use. This gesture is generated by collecting the user’s bone nodes.

#### 3.3.2. Posture Correction Module

The Posture Correction Module mainly uses the DTW algorithm [40] to make a dynamic comparison and analysis of body position in order to satisfy the real time requirement, with normalization of skeleton data comparing and evaluating different skeleton models. Although the skeleton model provides 25 joints, authors have observed that only nine joints contribute to identifying a bad back position. In order to generate the DTW’s feature vector, these joints (head, neck, left_shoulder, spine_shoulder, right_shoulder, spine_middle, left_hip, base_spine, and right_hip) are taken into account.

##### DTW Algorithm

The DTW algorithm is a dynamic programming algorithm which was initially used for speech recognition. The DTW algorithm makes similarity comparison between two sequences (time-correlated) possible. The length of the two sequences may not be the same, while the interval period for sampling the two sequences must be the same. The main idea is to eliminate the difference between the two different lengths of time, using a non-linear regularization function to model the volatility on the timeline by changing one of the timelines as much as possible with the addition of a timeline of coincidence.

We define feature space as F, original sequence as X (x_1_,x_2_,…,x_n_) and length as *n*; the user’s sequence during gameplay is Y (y_1_,y_2_,…,y_m_) and length *m*-in order to compare a set of features x and y, defining an overhead function c, *i.e.*, the distance between two sequences. A gesture sequence is the concatenation of such feature vectors.

Generally, the more similar x and y are, the lower the value of c(x,y) is. On the contrary, the shorter the distance between the two feature vectors is, the less similar x and y are, and the greater the value of c(x,y) is. The more distant the vectors are, the more overhead it is. If each c for each feature vector is obtained (x_n_,y_n_) (where c with n ∈ [1:N], m ∈ [1:M]), then an overhead matrix C can be obtained, of which C(n,m) = c(x_n_,y_m_). Similarity comparison between the two sequences can be transferred into finding the shortest path between X and Y in overhead matrix C.

According to the theory of the shortest path, the optimal path P* between X and Y is the shortest path among all possible ones, in which case the DTW distance between X and Y is:

DTW(X,Y) = cp*(X,Y) = min{cp(X,Y)|p is a (N, M) regular path}(1)

The Posture Correction Module uses DTW(X, Y) to obtain its value, and is then converted into an error or non-error data and sent to the Feedback module.

The Posture Correction Module is constantly recording the user’s movements during the gameplay. Whenever a gesture is made, that is: bad posture is adopted (error data), the game alerts the user, indicating that his back is in an incorrect posture.

##### Node Data Normalization

Since the stored original posture is not consistent with that of the user, the distance between body and Kinect device may also lead to various skeleton data, even for the same person. Normalization of human skeleton node information was needed in order to fit it into the DTW algorithm.

Taking the shoulder center node of the skeleton model as a reference, the distance from the left shoulder to the right one is standard since it is relatively fixed, and hence the skeleton data normalization formula can be defined as:

(2) $${\vec{P}}_{\;norm}=\frac{\vec{P}-\vec{C}}{\left|\vec{L}-\vec{R}\right|}$$

Among them, *P* is the vector before being normalized and *C* is the vector of the shoulder center corresponding to the world coordinate system before being normalized. *P_norm_* is the vector after being normalized. *L* and *R* are vectors for the left shoulder and right shoulder respectively, and normalization of the skeleton data can thus be achieved in this way.

The Posture Correction Module is continuously reading the skeleton joints of the user and passes the skeletal data to the DTW processor. This function is used to transform the data of the skeleton in a more useful format for DTW by extracting the coordinates and normalizing them. After this, the DTW algorithm checks if the given sequence is a gesture by assuming that the gesture ends on the last observation of the sequence.

#### 3.3.3. Feedback module

The feedback module emits an alert during the gameplay, indicating to the user that distance and tilt need to be corrected once a deviation has been detected. This deviation is detected after completing a gesture, *i.e.*, a body deviation of more than 20 degrees with respect to the back’s original vertical position, as much to the right as to the left. This gesture is recorded before starting the game. After a gesture has been made, an error message is sent to the feedback module. This module displays an alert on the screen and stores the number of errors made and the time in which they occur (actual time). All this information is stored in the users’ profile with the total time needed to accomplish the levels. The physiotherapists can follow the user’s progress with this information.

This posture correction system is included inside the virtual game which is explained below.

### 3.4. Game System Overview

Games are not usually designed with age-related impairments in mind, which has a negative impact on elderly users. For this reason, the game presented in this paper has been developed taking into account the possible physical and cognitive age-related disabilities and end-users’ experiences. The entire design process of the game is described in a previous work [41] by the authors. Game design guidelines described in Gerling *et al.* [42] have also been taken into account in order to adapt the game to the players’ motor skill level. For instance, a large number of gestures do not have to be memorized by the users, and in order to avoid fatigue, game difficulty adjustments are provided to them.

To implement the game environment, Unity 5.2, a professional 3D game engine offering a free license was used as a development tool in order to make the users’ experience more realistic.

The depth information about the scene is represented using a grey-scale at the bottom part of the screen: the darker a pixel, the closer it is to the camera. The pixels represented in black correspond to those without any depth information; this usually happens when the objects are too close or too far away from the camera.

The primary goal of this game is to perform physical exercises by having fun, while the posture correction system prevents users from adopting incorrect postures that are not ideal for their physical function.

Whenever the game starts, the process is as follows:

1. At the beginning of the game, users will be asked to enter their name, which will be referenced in the scores in order to store any related information during the gameplay. Also, the *settings* options can be configured.
2. Then, the First Recognition Module explained above is run, and the user’s original posture is stored.
3. The game starts with the first level and the user must collect all the objects appearing on the screen. Here, the Posture Correction Module is running and checks continuously whether the user posture deviates from the original one. If this action takes place, the game subtracts points from the total, obtaining more points if the object has been collected using correct posture.

The game comprises three levels, the aim of which is for the objects that appear not to fall to the ground. These objects–cakes and bottles of wine–appear on the screen following both horizontal and vertical paths, thus ensuring that the user has to perform specific movements, as defined by the physiotherapists, to collect each of them. The movement consists of shoulder abductions in order to collect the objects and shoulder adductions whenever the user needs to rest his arm. The exercise involves raising the arm (abducting) along the sagittal plane up to game object level while keeping the elbow locked, and then lowering (adducting) the arm back down to the user’s side. In this way, users need to perform physical movements with their arms to collect the objects and, in turn, stimulate the cognitive area. Each level lasts three minutes so as to prevent users from getting tired, although this duration can be adjusted by the physiotherapists to avoid fatigue in training. At the first level, the objects follow a vertical path. At the second level, the number of these objects increases and at level three the objects follow a horizontal path. A piece of cake is awarded on completion of each level until the whole cake is obtained after completing the third and last level.

Furthermore, the game provides a settings menu in order to make the game more widely usable. In this menu the type of user can be specified taking into account that users may have limited mobility in either arms (even absence of absolute movement in either of the two limbs) or in their legs (use of wheelchair). In this case, the users may choose if they wish to play with their left arm, right arm, or both or even select the option to play standing or seated. With this information, the game changes its behaviour to make it easier.

Users might then have to calibrate the sensor manually in order to check their position (1.5 m from the sensor). In this case, the menu offers users the option of checking their body by watching their avatars on an empty screen. The primary goal here is to have the complete human figure inside the computer screen. Users should check the sensors’ field of view (by moving the sensor manually up and down) until the avatar is seen inside the screen.

One of the important aspects of this work is to highlight the difficulties that some users faced to calibrate the Kinect sensor before starting the experiment. Calibration is an important and essential step for the Kinect to be able to track and interpret users’ movements and if it is not done properly, the device might not read the whole body, thus making it impossible to play.

With this customized settings menu, physiotherapists can adjust movements according to the individual user’s conditions, and have greater flexibility to adjust their training physiotherapy programs to users’ specific needs.

**Figure 3:**
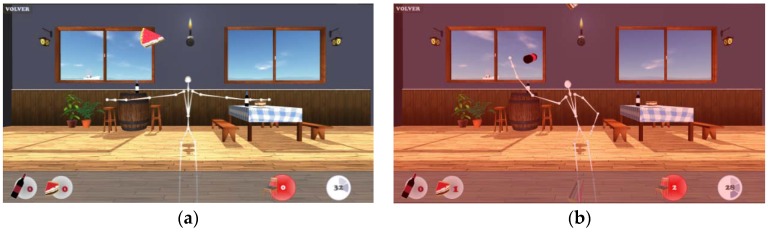
(**a**) Screen of a user playing correctly; (**b**) Screenshot of an error while playing incorrectly.

In order to enhance the users’ game experience, the game is played in a 3D environment in which the bottles and cakes fall to the floor (seen Figure 3a). Every level has a different background picture of the environment and players can see their individual arousal score indicated below, as well as the time remaining and errors made. In order to collect an object the player must make an arm movement to reach it. If the player chooses the right hand mode to play, the objects will only appear on the right side of the screen and vice-versa.

Users see themselves on the screen interacting with the virtual story in real time, using trunk and limb movements, so that it appears as though the user is part of the virtual environment.

If body position fails to keep within the fixed range of movement, *i.e.*, the user completes a gesture, an error score appears on the bottom part of the screen and as feedback, a red explosion appears on the screen (see Figure 3b). This message provides information about the degrees of tilt and head distance to be corrected by the user. After an error, the user loses two points from the total score.

A series of control parameters have also been established and which are gathered during the course of the game. These parameters are: the time set per level, the amount and speed of the objects that appear on the screen and the possibility of changing their paths, thus enabling different movements to be performed with the arms. Depending on each user’s characteristics, these parameters can be modified from the set-up menu to make certain levels easier if necessary. If the user considers the game to be too easy, the physiotherapists can change the values of these parameters to make the game session more difficult.

During gameplay, feedback (collected items, scores… *etc*.) is provided to the user as well as visual, auditory, and textual feedback on the users’ performance. After the game session, physiotherapists and users can take a look at the data stored in their profile to determine the user’s progress. All this information has been stored during the gameplay in the Feedback module.

## 4. Results

Participants’ demographic details and test results were analyzed using SPSS v22. In this section the results of each test are described.

As previously mentioned, experts collected data during the game sessions. These data refer to the number of incorrect postures adopted by the users during the gameplay. See Table 2.

In some cases, the Kinect device failed to obtain an accurate depth value and skeleton model, originating an incorrect detection of the complete posture. The overall successful posture recognition percentage was 95.20%.

Furthermore, the data obtained after performing the two game sessions (P1 and P2) comprised some features such as: the number of objects collected (cupcakes = Object1 and bottles of wine = Object2), the number of errors produced due to bad body inclination and the users’ tilt degrees, that is, the original body position that is recorded during the First Recognition Module.

In order to perform an inferential analysis, first a normality test was conducted, through the Shapiro–Wilk test, considering that the participants sample was small (*n* < 30).

The results obtained show that a significant difference exists in the results obtained from the objects collected in the first session (*p* = 0.180) and in session 2 (*p* = 0.649); and the number of errors (*p* = 0.134 and *p* = 0.624) for both first and second sessions, but no significant difference exists in the user’s degrees tilt.

In a further analysis, a Mann-Whitney test was used to find differences between male and female participants. This test concluded with no differences in terms of results or in terms of participants’ age.

In addition, a nonparametric test for two related samples (Wilcoxon signed-rank test) was conducted (see Table 3).

Overall, the data provided positive ranks between the objects collected obtained during the first game session and the second session, *i.e.*, more objects were collected in the last one; and negative ranks were obtained between the number of errors during the first and second sessions, that is, the number of errors decreased in the second session.

Regarding the tilt degrees, 14 participants showed no changes after performing the two sessions. However, we will take into account that one participant’s results indicate a reduction in the tilt degrees. This, though far from proving anything, suggests that it may result in gradual improvement if the game is used for a long period of time.

In order to make some correlations between the data obtained previously, the Spearman Rank-order correlation analysis was conducted.

**Figure 4:**
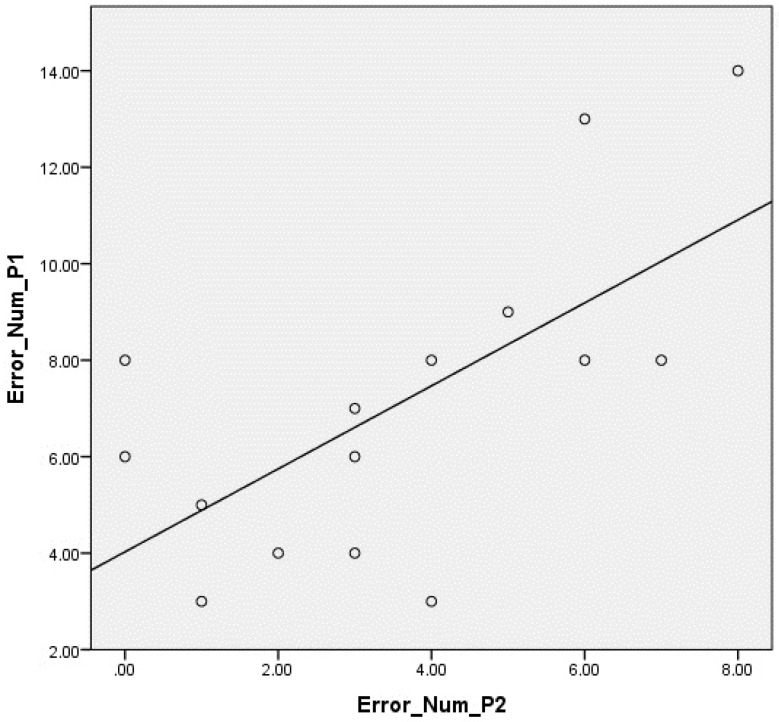
Correlation graph between the number of errors during the first and second sessions.

A correlation was found between the Number of Errors from the first game session and the second one (*r* = 0.608, *p* = 0.16). After six months playing, participants made fewer errors than during the first game session. Even when a player made a lower number of errors in the first session, the number in the second one decreases. This correlation may show that the more the users play, the more they learn how to play the game in a correct postural position. The correlation can be seen in Figure 4.

### Game Usability Scale Questionnaire

To measure game acceptance and its usability, the authors have used a modified system usability scale (SUS) [43]. This scale consists of 10 items with a score ranging from 0 to 100. According to the pilot study by Nacke, Schild, and Niesenhaus [44], the authors also altered this scale by replacing some words.

After the subjects played the game for two sessions, this modified SUS was used to assess the usability of the game. This questionnaire is a highly-regarded assessment tool, being both robust and reliable, as there is a correlation between subjective usability measures.

According to Tullis and Albert [45], an average SUS score under 60% is relatively poor and one over 80% can be considered good.

Regarding the results obtained from all the questionnaires, even when the scores for the first session were good (M = 74.64, SD = 4.25), the average SUS score after the second game session can be considered to be very good (M = 86.78, SD = 2.85).

## 5. Conclusions and Further Research

A physical activity intervention exergame to prevent the elderly from making incorrect postures while playing has been presented in this paper.

The main aim of the research has been to develop a game for elderly persons that detects incorrect postures, thus preventing them from playing badly and acquiring bad habits.

The game was approved and tested using a diverse group of elderly persons from a nursing home and the application implemented successfully tracked body positions and orientations of all the participants. The system detected incorrect postures by implementing the DTW algorithm during the posture detection.

The virtual game’s validity using the Kinect 2.0 device was demonstrated following analysis of the results shown previously. The system was evaluated with 15 users, achieving a successful posture recognition percentage of 95.20%.

Regarding the results obtained, the tests conducted concluded with positive rank in the objects collected and negative in the errors made, *i.e.*, after six months playing, users collected more objects and made fewer errors, achieving better learning about their postural control. Additionally, the results show that there were no differences either in sex or age for the selected participants.

From this solid foundation, Kinect-based gaming has significant potential to create a low-cost and enjoyable exercise setting while simultaneously gathering quantitative data related to the correct or incorrect postures of the users.

The system makes use of the Kinect sensor both to play and detect incorrect postures during the game. This sensor enables the user to interact with the game by making physical movements, and this natural method of human-computer interaction allows for the development of specialized forms of elderly care applications.

This exergame was developed initially to be played in a nursing home due to the competitiveness that games provide between players, but it may be possible to target people who live independently at home.

In the future, researchers will carry out a long-term study to ascertain whether participants can, after playing over a longer period of time, try to project what they have learned and correct their posture in their daily lives owing to the error feedback provided by the game.

## Figures and Tables

**Table 1:** Participants listed in the order in which they were recruited showing their age, sex, diagnoses, and MMSE levels and scores.

No. Part.	Age	Sex	Disability	MMSE Level	MMSE Score
1	84	M	Muscular Dystrophy	Mild	22
2	77	M	Macular degeneration	Normal	26
3	69	M	Macular degeneration	Normal	27
4	85	M	None	Mild	20
5	74	M	None	Normal	24
6	82	F	None	Normal	25
7	89	F	Presbyopia	Mild	21
8	96	F	None	Normal	26
9	73	F	Muscular Dystrophy	Normal	27
10	87	F	Parkinson	Moderate	19
11	84	F	None	Normal	25
12	94	F	Presbyopia	Mild	20
13	87	F	Muscular Dystrophy	Normal	24
14	85	F	None	Normal	27
15	88	F	None	Normal	24

**Table 2:** Mean performance of the posture recognition system for both sessions.

No. Part.	Incorrect Postures Mean	Mean of Incorrect Postures Detected	Mean of Incorrect Postures Not Detected
1	4	4	0
2	9.5	9.5	0
3	5	5	0
4	12	11	1
5	7	7	0
6	2	2	0
7	6	6	0
8	8	7	1
9	4.5	4.5	0
10	3.5	3.5	0
11	3	3	0
12	9	7.5	1.5
13	4	3.5	0.5
14	3	3	0
15	3	3	0
Total	83.5	79.5 (95.20%)	4 (4.79%)

**Table 3:** Wilcoxon signed ranks test statistic.

Num_Object1_P2 and Num_Object1_P1	Num_Object2_P2 and Num_Object2_P1	Num_Errores_P2 and Num_Errores_P1	Tilt_P2 and Tilt_P1
Z = −2.370	Z = −2.422	Z = −3.307	Z = −1.000
*p* = 0.018	*p* = 0.015	*p* = 0.001	*p* = 0.317
